# Bayesian central statistical monitoring using finite mixture models in multicenter clinical trials

**DOI:** 10.1016/j.conctc.2020.100566

**Published:** 2020-04-09

**Authors:** Tomoyoshi Hatayama, Seiichi Yasui

**Affiliations:** a Biostatistics Department, Quantitative Sciences Division, R&D, Janssen Pharmaceutical K.K., Tokyo, Japan; b Department of Industrial Administration, Faculty of Science and Engineering, Tokyo University of Science, Chiba, Japan

**Keywords:** Risk-based monitoring, Posterior predictive distribution, Abnormality detection, Outlier detection, Contamination

## Abstract

**Background:**

Central monitoring (CM), in which data across all clinical sites are monitored, has an important role in risk-based monitoring. Several statistical methods have been proposed to compare patient outcomes among the sites for detecting atypical sites that have different trends in observed data. These methods assume that the number of clinical sites is not small, e.g., 100 or more. In addition, the proportion of atypical sites is assumed to be relatively small. However, in actuality, the central statistical monitoring (CSM) has to be implemented in small or moderate sized clinical trials such as small phase II clinical trials. The number of sites is no longer large in such situations. Therefore, it is of concern that existing methods may not work efficiently in CM of small or moderate sized clinical trials. In the light of this problem, we propose a Bayesian CSM method to detect atypical sites as the robust method against the existence of atypical sites.

**Methods:**

We use Bayesian finite mixture models (FMM) to model patient outcome values of both atypical and typical sites. In the method, the distributions of outcome values in normal sites are determined by choosing the body distribution, which has the largest mixture parameter value of finite mixture models based on the assumption that normal sites are in the majority. Atypical sites are detected by the criterion based on the posterior predictive distribution of normal site's outcome values derived from only the chosen body distribution.

**Results:**

Proposed method is evaluated by cumulative detection probability and type I error averaged over sites every round of CSM under the various scenarios, being compared with the conventional type analysis. If the total number of patients enrolled is 48, the proposed method is superior at least 10% for any shift sizes at the 2nd and the 3rd rounds. If the total number of patients is 96, both methods show similar detection probability for only one atypical site and large shift size. However, the proposed method is superior for the other scenarios. It is observed that all the type I errors averaged over sites are little difference between the methods at all the scenarios.

**Conclusion:**

We propose a Bayesian CSM method which works efficiently in a practical use of CM. It is shown that our method detects atypical sites with high probability regardless of the proportion of the atypical sites under the small clinical trial settings which is the target of our proposed method.

## 1 Introduction

The role of monitoring activity in clinical trials is to protect patients participating in clinical trials, to confirm that the operation of the trials is complying with protocols and regulatory requirements, and to ensure the accuracy and completeness of reported data [1]. In recent years, however, the cost of monitoring activities has been increasing with complicating clinical trials, and their operating cost has become a large proportion of whole cost. It is, hence, necessary to improve the efficiency of monitoring activities [2]. So far, monitoring with frequent visits to clinical sites and 100% source data verification (SDV) has been conducted. This approach, however, has crucial limitations on quality control of clinical trials, which is that data from the relevant site cannot be compared with data from other sites. Thus, it does not contribute the improvement of data quality. Currently, the 100% SDV is not considered cost-effective [[2], [3], [4], [5]]. Nowadays, by advanced electronic systems such as electric data capture systems, it is possible to review data without visiting sites. Consequently, data from multiple sites has been reviewed centrally, which is known as central monitoring (CM). Thus, abnormalities on operational processes can be detected by CM, and the sites to perform on-site monitoring are efficiently identified [6,7]. The implementation of the CM is recommended by Food and Drug Administration (FDA) [6].

It is useful to apply statistical methods to CM in order to detect abnormalities on operational processes effectively, and a statistical method for central statistical monitoring (CSM) has been proposed [[7], [8], [9], [10], [11], [12]]. Most of the researches on CSM are aimed at detecting fraud. In recent years, however, several methods have been proposed to compare outcomes among the sites in order to detect atypical sites with potential abnormalities on operational processes, thereby the sites to be visited are clarified [7,13]. As shown in Venet et al. [7] as a principle of CSM, reported data are collected based on a common protocol and a case report form for clinical trials. Thus, even if the clinical trial is for the case of multicenter clinical trials, outcomes from one site should has basically similar trend to other sites. Therefore, the sites which have the different outcome tendencies should be detected as atypical sites, and it is able to be recognize that abnormality on the operational process may have occurred in the sites. Thus, it is reasonable to identify the atypical sites to implement efficient on-site monitoring and inspection.

In a decade, several methods for CSM have been proposed, which are classified into two types that are so-called supervised analysis and unsupervised analysis [9]. The supervised analysis is grading of sites by a key risk indicator (KRI). The unsupervised analysis is identification of outlier sites. The unsupervised analysis is useful to achieve efficient on-site monitoring and inspection. Thus, our study focuses on the unsupervised analysis, i.e. finding atypical sites identified as outliers.

Most of procedures based on the unsupervised analysis are descriptive statistics (histogram, volcano plot, etc.) and techniques based on the basic theory of statistical inference (the chi-square test for the Mahalanobis distance), which are summarized in Oba et al. [9]. Spiegelhalter [14] has proposed the analysis of the incidence of events, e.g. binomial variates, Poisson counts, using a Funnel plot in order to compare clinical sites, which is developed by modifying the concept of control charts which was introduced in statistical quality control for manufacturing. Desmet et al. [15] has proposed an analysis method for continuous data (normal variates) using linear mixed effects models to detect atypical sites. Desmet et al. [16] proposed the method analyzing the incidence of events using Beta-Binomial Models to detect atypical sites. For detection using multiple outcomes, currently the atypical sites are detected as multivariate outliers by the chi-square test based on the Mahalanobis distance [9]. After detection, though why these sites are atypical are investigated, it is necessary to specify how the individual variables contribute to each multivariate outlier. Mason et al. [17] and Zink et al. [18] tackle this problem. For this problem, Zink et al. [18] illustrated the application of the contribution plot [19] to RBM for real multicenter clinical trials. Mason et al. [17] proposed to calculate the magnitude of the contribution using decomposition of Hotelling's *T*^2^ statistic into one dimensional variate.

These methods have been developed in the main aspect to good quality management of global clinical trials. In unsupervised procedures, hence, it is assumed that the number of clinical sites is not small, e.g., 100 or more. In addition, the proportion of atypical sites is assumed to be relatively small, e.g., 1% or 5% because the target of such procedures is outlier detection. However, in actuality, the CSM has to be implemented in smaller sized clinical trials such as small phase II clinical trials. The number of sites is no longer large in such situations. In addition, because CSM is conducted during the mid-period of clinical trials, for the early term that all of the clinical sites have not been opened yet, and the analysis is conducted in a small number of clinical sites. Hence, in these clinical trials, the proportion of the atypical sites is not relatively small. In the situations where the total number of clinical sites is small in the early period, e.g., 10, the existence of one abnormal site makes relatively large impact to the proportion of abnormal sites. In those cases, it is difficult to detect atypical sites with high probability by conventional methods. It is, therefore, necessary to develop a CSM method for smaller clinical trials or the beginning of clinical trials in such cases.

In order to decide the clinical sites that have to be visited in CSM, it is important to easily discriminate relevant sites from non-relevant sites by analyzing data tendency. Unfortunately, it is difficult to powerfully detect abnormal sites in clinical trials that we target. However, since the unsupervised CSM is outlier detection, it is natural that abnormal sites are assumed to be minority. Thus, it is useful to apply Bayesian statistics to CSM which is able to combine, because posterior probabilities are able to express the possibility of abnormality of an operational process, which shows the magnitude how an outlier is crucial in terms of undesired impact to the goodness of a clinical trial.

In actual clinical trials, CSM is conducted multiple times, e.g. typically monthly [20], during the trials in order to detect abnormal operational processes. Multiple CSM is PDCA (Plan-Do-Check-Action) cycle, which originally is process control for quality control in manufacturing. It contributes to promptly finding potential data quality risks and maintaining stable operational processes by finding root causes using problem solving methods and corrective actions. Therefore, iterative feature should be incorporated with the statistical methods for CSM, and such methods should be tested in the situation under which the multiple statistical analyses are conducted. In fact, the control charts, which are definitive tools for statistical process control, are evaluated by the average run length which is the mean time to the first occurrence of out-of-control signal [21]. Furthermore, Exponentially Weighted Moving Average (EWMA) control charts and CUmulative Sum (CUSUM) control charts are representative control charts in which whether the current state of a process is in-control or out-of-control is judged using accumulated information of the past process states [21]. However, existing CSM methods have not been sufficiently developed and evaluated under such situations.

EWMA and CUSUM control charts have more powerful performance to detect abnormality. Thus, the concept of the accumulation is useful in our targeting clinical trials because the number of data is small. However, the data accumulation generates a contaminated distribution if there are abnormal sites in a clinical trial, and the simple accumulation procedures suffer from declined power. Therefore, in this study, we propose a Bayesian central statistical monitoring method using Bayesian finite mixture models [[22], [23], [24]] to detect atypical sites with avoiding contamination in smaller clinical trials or the early phases of clinical trials of which size is assumed in conventional CSM. Our method is evaluated in the cases where multiple CSM is conducted during the trials.

In the next section, we outline CSM in clinical trials and indicate impact of data from abnormal clinical sites on CSM performance. In Section 3 we propose a CSM method using Bayesian finite mixture models. We present simulation studies to examine the operation characteristics of a new method in Section 4, and we explain our proposed method in detail that what data are used in each analysis and how to make decision using artificial example in Section 5, followed by discussion.

## 2 CSM and statistical problem on detecting atypical sites

### 2.1 Implementation of CSM

In this section, we briefly introduce CSM and describe how outcomes from atypical sites affect the lower performance of CSM. In this study, we make a simple assumption to clarify our proposal that outcomes are continuous variables observed once in a trial. All the outcomes are assumed to follow independent and identical normal distributions. However, the means of outcomes from atypical sites are shifted. Hence, the purpose of our study is to develop the statistical detection technique for the atypical sites, which have their mean shifted normal distributions.

**Fig. 1:**
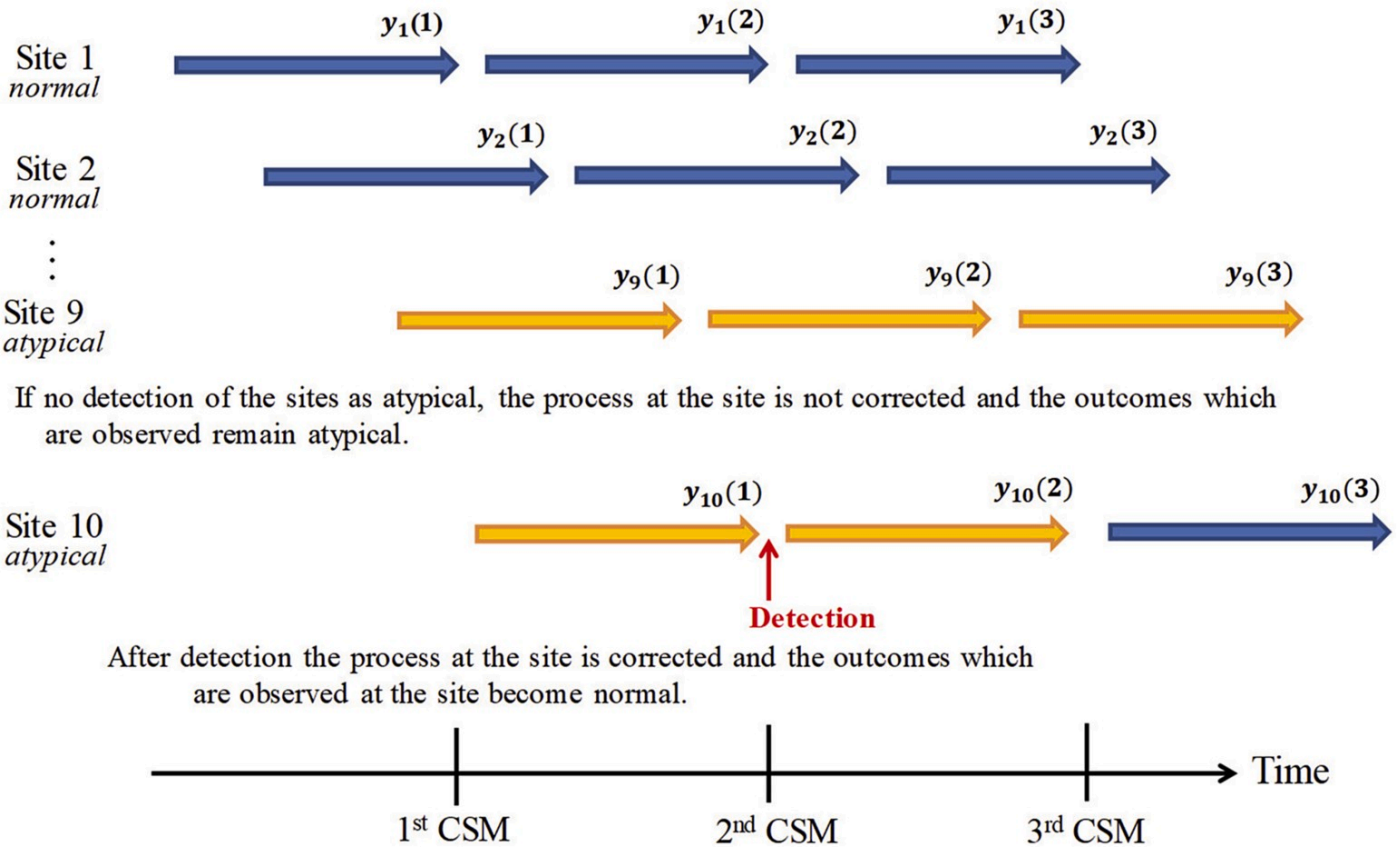
Atypical sites detection and process correction.

Here, we assume clinical trials in which CSM is conducted multiple times. Let $y_{ij}$ be the outcome of the j-th patient in the i-th site, and M (M≥2) sites take part in a clinical trial. $Y_{i}\left(t\right)={\left(y_{i1}\;y_{i2},\ldots ,y_{iN_{i}\left(t\right)}\right)}^{'}$ is the data vector of which the elements are all the outcomes that have been observed in the i-th site by the t-th (t=1,…,T) CSM, where $N_{i}\left(t\right)$is the total number of patients enrolled to the i-th sites by t-th CSM. The atypical sites are detected by comparing outcomes of the site with those of other sites. However, it should be note that only process abnormality is not necessarily detected by comparing outcomes between sites. The differences in patient population characteristics such as ages, races and severity among sites, or differences in the selection of concomitant therapy are possibly detected. In general, these differences occur by systematic causes and should be adjusted as covariates if these are identified in advance. Fig. 1 shows the relationship between the detection of atypical sites by CSM and the change of distributions of outcomes as a conceptual diagram. The sites are opened as a staggered manner in practical clinical trials. In the early round of CSM, small proportion of sites may be analyzed in the CSM because the analysis is performed using available data at the timing of the CSM. In the early round of the CSM, it is particularly difficult to conduct CSM effectively due to small amount of the data, and in the case, the harmful effect of the data obtained from atypical sites to the statistical inference is to be relatively large. If atypical sites are detected by CSM, the atypical sites are investigated by on-site monitoring. Then, the root causes in the processes of those sites are explored and the corrective actions for the causes are implemented. After the corrective action, the processes of the atypical sites recover normal status, and normal outcomes are observed in those sites. Thus, the overall mean of all the observed data is not useful to detect atypical sites in monitoring of means of sites. The t-th round of CSM analysis that whether the i-th site is normal or not should be implemented based on the latest outcome that have been observed for the period after the (t−1)-th CSM. Hence, the data analyzed at the t-th round of CSM analysis for the i-th site is denoted as $y_{i}\left(t\right)\;\left(y_{i}\left(t\right)\;=Y_{i}\left(t\right)\backslash Y_{i}\left(t-1\right)\;,\;\;t>1,\;\;\;y_{i}\left(1\right)=Y_{i}\left(1\right)\;\;\;t=1\right)$, where $Y_{i}\left(t\right)\backslash Y_{i}\left(t-1\right)$ is the relative complement of $Y_{i}\left(t-1\right)$ in $Y_{i}\left(t\right)$. To conduct CSM effectively, the initiation timing of the CSM should depend on how much sites are opened and how first data is accumulated.

### 2.2 Statistical problem of CSM based on single distribution

The conventional CSM procedures focus on relatively large clinical trials. The detection techniques to find atypical sites using the simple statistical model based on a single distribution is useful, because it is reasonable to implicitly assume the relatively small ratio of atypical sites for normal sites [[14], [15], [16]]. In actual, however, the smaller sized clinical trials have been conducted as well. In smaller sized clinical trials, the early phase such as the small Phase II clinical trials or the beginning of a clinical trial, the ratio of atypical sites for normal sites are relatively bigger if atypical sites exist. Hence, these conventional procedures work less effectively in our situation. Desmet et al. [15] analytically described the influence that the ratio of atypical sites affects the detection performance in detecting location shifts under the normal distribution assumption. Desmet et al. [15] showed that the detection performance is deteriorated if more than 10% of all the sites are atypical and that the hypothesis testing to detect atypical sites is not an unbiased test if more than 30% of all sites are atypical. In the next paragraph, we qualitatively describe the impact that data from atypical sites affecting the detection procedure in which the single distribution is assumed as the statistical model of all data. In this study, outcomes are normal distributed variable including laboratory values of HbA1c and blood pressure value as example for simpler discussion. It is considered that the causes of variation of these variables are to misunderstand the interpretation of the study protocol in the sites and to be inconsistencies in the medication guidance, the measurement method or settings of measurement equipment. The causes generate systematic differences on these values in naturals. Though various types of variables are analyzed in CSM [8,9], we focus on a CSM technique for normal variates as a basic approach. Furthermore, the treatment for other kinds of variables is mentioned in the discussion section.

The outcomes $y_{ij}$ for patients enrolled in normal sites have the normal distribution $\;N\left(\mu ,\sigma^{2}\right)$ . The outcomes $y_{ij}$ for patients enrolled in atypical sites have the normal distribution $\;N\left(\mu +\Delta ,\sigma^{2}\right).$ If outcomes from atypical sites are included in all the analytical data, the outcomes from normal sites are contaminated. If we denote data distributions as a single distribution constructed by matching both the first and the second moments with those of data distribution, the statistical model is

(1) $$y_{ij}\;\sim \;N\left(\mu +r\Delta ,\;\sigma^{2}+r\left(1-r\right)\Delta^{2}\sigma^{2}\right),$$

where r(0<r≤1) is the ratio of patients observed from atypical sites for patients of all the sites. If outcomes of $N_{i}\left(t\right)$ patients in the i-th site are obtained by the t-th CSM, the statistical model of the average ${\bar{y}}_{i}\left(t\right)$ of outcomes on the i-th site is

(2) $$\;\;\;\;\;{\bar{y}}_{i}\left(t\right)\;\sim \;N\left(\mu +r\Delta ,\;\frac{\sigma^{2}+r\left(1-r\right)\Delta^{2}\sigma^{2}}{N_{i}\left(t\right)}\right)\;,$$

where ${\bar{y}}_{i}\left(t\right)=\sum_{j=1}^{N_{i}\left(t\right)}y_{ij}$. From this formula, we are able to directly understand as follows: if we adopt a single distribution to a statistical model, the mean of the distribution for site average ${\bar{y}}_{i}\left(t\right)$ is affected by data obtained from atypical sites, as a result, the distribution of the site average ${\bar{y}}_{i}\left(t\right)$ shifts to the distribution for atypical sites. In addition, the variance of the site average ${\bar{y}}_{i}\left(t\right)$ increases by factors r and $\Delta^{2}$.

**Fig. 2:**
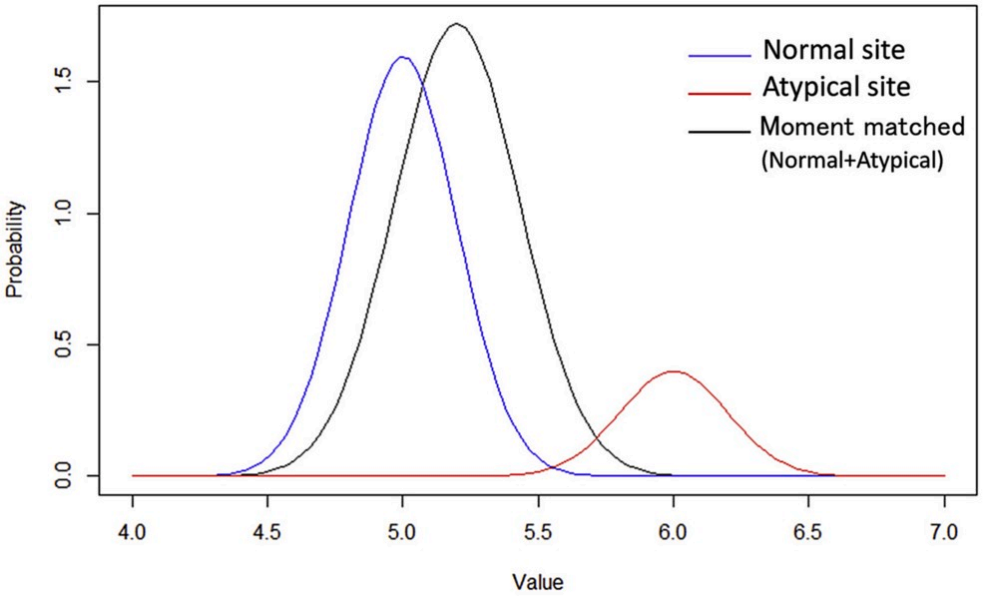
The patient outcome distributions for the case of contaminating data with 20% atypical site data.

For instance, we consider the case in which atypical sites are 20% of all the sites. The site averages independently follow identical $N\left(0,1^{2}\right)$ in normal sites, and the site averages for atypical sites independently follow identical $N\left(1,1^{2}\right)$. Five patients are assumed to be enrolled in each site. Then, the site averages for normal sites have N(0,0.2) and the site averages for atypical sites have N(1,0.2). The formula (2) is N(0.2,0.232) in this case. In Fig. 2, the distributions of site averages from normal sites and atypical sites are shown by the blue and red solid lines, respectively. The detection procedures based on the single distribution use the contaminated distribution expressed by the black solid line in Fig. 2. The distribution of an overall average (black solid line) largely overlaps with the distribution of the site average for atypical sites. Conventional procedures proposed the CSM to detect atypical sites with criterion if the site average is out of thresholds determined on the simple distribution model (2), e.g., the 5th percentile and/or the 95th percentile, then the site is found to be atypical. It is clear that those procedures are less effective unless the r is extremely small.

Consequently, it is necessary to develop the statistical procedure which does not depend on the ratio r of atypical sites and is applicable in small sample size situations.

## 3 Bayesian CSM

We propose a finite mixture model (FMM) approach to detect atypical sites in CSM. The whole data generates from the mixture distribution whose components are the distributions for normal and atypical sites. This approach mitigates the effect of the ratio r of atypical sites. Thus, the power of detection of this approach is expected to be higher. If considering that the sites participating in clinical trials are regularized, it is natural to assume that the normal sites are a majority. We focus on the unsupervised approach of CSM which attempts to identify problematic sites as outliers. This approach is based on the concept of the pareto principle and is the one of the most important principles to perform efficient process control in quality management [25]. Hence, we should find a few sites with strange tendency from many of sites at the first priority. In other words, the component distribution with the highest mixture weight corresponds to be normal sites. After estimating all the parameters of FMM by Bayesian inference, only the estimated majority distribution is used to detect atypical sites. The atypical sites are detected by comparing the site average of outcomes with the estimated majority distribution.

In section 3.1, we introduce the Bayesian detection method based on single distribution as the conventional procedures. This method is called the central statistical monitoring method based on single distribution and it is shortened as single CSM. In Section 3.2, we develop the Bayesian CSM method based on Finite Mixture Model (FMM), and we call it the central statistical monitoring method using Bayesian finite mixture models, it is abbreviated as FMM-CSM.

### 3.1 CSM method based on single distribution

We introduce a single CSM method based on a conventional concept. In the method, the statistical model of observed outcomes Y(t) until the t-th round of CSM is

(3) $$h\left(Y\left(t\right)|\;\theta \right)=\prod_{i=1}^{M}\prod_{j=1}^{N_{i}\left(t\right)}f\left(y_{ij}|\theta \right),$$

where $f\left(y_{ij}|\theta \right)$ is the density function of the normal distribution with parameters θ=(μ,σ) in which μ is the expectation and σ is standard deviation, prior distributions of μ and $\sigma^{2}$ are $N\left(\mu_{0},\sigma_{0}^{2}\right)$ and InvGamma(a,b) respectively. InvGamma(a,b) denotes the inverse-Gamma distribution with the positive valued parameters a,b.

The posterior predictive distributions $p\left({\bar{y}}_{i}\left(t\right)|Y\left(t\right)\;\right)$ of site averages ${\bar{y}}_{i}\left(t\right)$, i.e., the average of elements of the vector $y_{i}\left(t\right)$ is calculated as follows:

(4) $$p\left({\bar{y}}_{i}\left(t\right)|Y\left(t\right)\right)=\int^{\;}f\left({\bar{y}}_{i}\left(t\right)|\theta \right)p\left(\theta |Y\left(t\right)\right)d\theta ,$$

Based on Eq. (4), we identify that the i-th site is an atypical site if the site average for the i-th site at the t-th round of CSM satisfies the following condition;

(5) $${\bar{y}}_{i}\left(t\right)<\lambda_{i}^{\alpha }\left(t\right)\;\;\;\;\;\;\text{or}\;\;\;\;\;\;{\bar{y}}_{i}\left(t\right)>\lambda_{i}^{1-\alpha }\left(t\right)\;\;,$$

where $\lambda_{i}^{\alpha }\left(t\right)$ is the 100α-th percentile of the $p\left({\bar{y}}_{i}\left(t\right)|Y\left(t\right)\right)$.

In this method, as explained in section 2.1, after the detection, corrective action to the sites are implemented. The normal outcomes are observed in those sites after these actions, all the available data are taken over into calculate the likelihood function and sample size has increased every round of CSM. Thus, the formula (4) is the posterior predictive distribution of a site average for each site. The actual site average ${\bar{y}}_{i}\left(t\right)$ is calculated from only the current data at *t*-th round of CSM. If the criterion (5) held, a site is found to be atypical.

### 3.2 CSM method based on FMM

We propose a FMM-CSM method to detect atypical sites which mitigates the effect of the ratio of atypical sites in participating sites. At first, as considering that data obtained from atypical sites are contaminated by data obtained from normal sites, the statistical model of observed outcomes Y(t) until the t-th CSM is

(6) $$\begin{array}{ccc} g\left(Y\left(t\right)|\theta \right) & = & \prod_{i=1}^{M}\prod_{j=1}^{N_{i}\left(t\right)}\left(\sum_{k=1}^{K}\pi_{k}f\left(y_{ij}|\theta_{k}\right)\right),\\ \left(\pi_{1},...,\pi_{k}\right) & ∼ & Dirichlet\left(\alpha_{1},...,\alpha_{k}\right) \end{array}$$

where $\pi_{k}$ (k=1,…,K) are mixture parameters, and $\;f\left(y_{ij}|\theta_{k}\right)$ is the density function of the normal distribution with parameters $\theta_{k}=\left(\mu_{k},\sigma_{k}\right)$ in which $\mu_{k}$'s are the expectations and $\sigma_{k}$'s are standard deviations, prior distributions of $\mu_{k}$'s and $\sigma_{k}$'s are $N\left(\mu_{0},\sigma_{0}^{2}\right)$ and InvGamma(a,b) respectively. $Dirichlet\left(\alpha_{1},\ldots ,\alpha_{K}\right)$ denotes the Dirichlet distribution with the positive valued parameters $\alpha_{1},\ldots ,\alpha_{K}$. In term of the determination of number of components K, for example, we reasonably choose the minimum component number such as three by which is able to take into account the possibility that location shift of data from atypical sites are occurred in both lower and upper sides in small clinical trials or the beginning of the trials. In addition, the parameters $\alpha_{1},\ldots ,\alpha_{K}$ should be determined by the proportions of atypical sites and normal sites according to the experience of the trials in the similar therapeutic area if we have the information. Even if there is no information about that, as the guideline for setting these parameters we are able to set these values based on the assumption that normal sites are a majority. In either case, it is recommended to examine the operation characteristics of the methods by simulation studies under various scenarios.

Next, we assume that the normal sites are a majority. We define that the component of the mixture distribution with the largest estimated mixture parameter in the posterior means of the mixture parameters $\pi_{k}$ is the distribution of outcomes for the normal sites. In other words, the distribution of outcomes for the normal sites is estimated as the component

$$k_{b}=\underset{k\in \left(1,\ldots ,K\right)}{arg\;max}E(\pi_{k}|Y\left(t\right)).$$

We call the estimated distribution for normal sites “body distribution”. The posterior predictive distributions $p_{k_{b}}\left({\bar{y}}_{i}\left(t\right)|Y\left(t\right)\right)$ of site averages ${\bar{y}}_{i}\left(t\right)$ for normal sites is calculated using the body distribution as follows:

(7) $$p_{k_{b}}\left({\bar{y}}_{i}\left(t\right)|Y\left(t\right)\right)=\int^{\;}f\left({\bar{y}}_{i}\left(t\right)|\theta_{k_{b}}\right)p(\theta_{k_{b}}|Y\left(t\right))d\theta_{k_{b}},$$

where $p(\theta_{k_{b}}|Y\left(t\right))$ is the posterior density function with the parameter $\theta_{k_{b}}$ of the body distribution. Based on Eq. (7), we identify that the i-th site is an atypical site if the site average for the i-th site at the t-th round of CSM satisfies the following condition;

(8) $${\bar{y}}_{i}\left(t\right)<\gamma_{i}^{\alpha }\left(t\right)\;\;\;\;\;\text{or}\;\;\;\;\;{\bar{y}}_{i}\left(t\right)>\gamma_{i}^{1-\alpha }\left(t\right)\;\;,$$

where $\gamma_{i}^{\alpha }\left(t\right)$ is the 100α-th percentile of the $p_{k_{b}}\left({\bar{y}}_{i}\left(t\right)|Y\left(t\right)\right)$.

In this method, same as single CSM method, all the available data are taken over into calculate the likelihood function and sample size has increased every round of CSM. Thus, the formula (7) is considered that the posterior predictive distribution of a site average for the i-th site if the site was normal, because the distribution is derived based on only the body distribution. The actual site average ${\bar{y}}_{i}\left(t\right)$ is calculated from only the current data at *t*-th round of CSM. If the criterion (8) held, a site is found to be atypical. In the method of FMM-CSM and Single CSM, the value of α is determined to have appropriately performance of detecting atypical sites and controlling family wise type I error which prevents ineffective on-site monitoring. In this article, we use 0.05 as the value of α for simple discussion in the comparison of the performance between methods. The determining α and controlling family wise type I error are discussed in the discussion section.

FMM is similar to another single distribution approach which specifies the distribution for normal sites by robust estimation for the mean and variance (or standard deviation) [15]. The famous robust estimators for the mean are the median, the trimmed mean, and the winsorized mean. The famous robust estimators for the standard deviation are the inter-quartile range and the median absolute deviation. However, it is required to predetermine the percentage of scraping extreme data if using the trimmed mean and inter-quartile range, and it is difficult to predetermine the adequate percentage so that the precision of estimation is better. If the ratio of atypical sites for all sites is small, the bias of the inter-quartile range is larger and the variance of the unbiased inter-quartile range is larger. On the other hand, it is possible for FMM with three components to estimate this percentage. In addition, if appropriately, Bayesian statistics takes a prior knowledge into the analysis of CSM regarding the percentage of outliers (atypical sites).

## 4 Simulation studies

To examine the performance of our proposed method as the CSM method for small clinical trials, we conduct extensive simulation studies with varying atypical sites proportion and the size of location shift in outcomes in the settings of the small clinical trials. We evaluate detection probability for atypical sites in various scenarios. In addition, CSM is multiply conducted in actual clinical trials. Therefore, we examine how much the frequency of multiple analysis influences the detection performance.

### 4.1 Simulation study design

Because of target of our proposed method, we use a hypothetical clinical trial with ten clinical sites in the simulation studies. In our simulation studies, CSM is conducted three times. We assume that the number of patient enrollments into each site follows the multinomial distribution with $p_{1}=\ldots =p_{10}=1/10$. We set two study sizes, N=48 and N=96, as the evaluated the number of patients at the final analysis of a small sized trial and a moderate sized trial, respectively. CSM is conducted each time of one third of patients is completed.

**Fig. 3:**
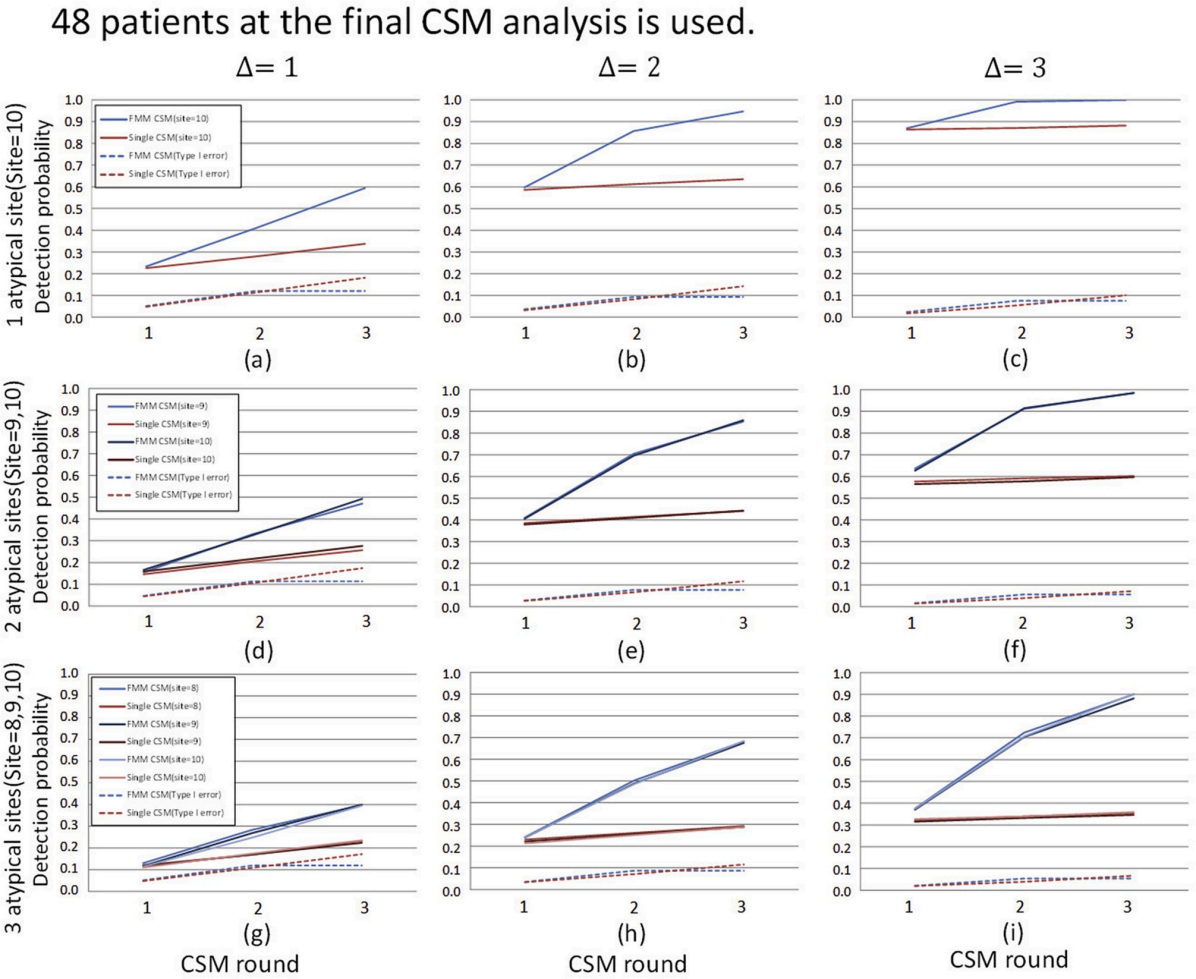
Cumulative detection probability stratified by location shifts and the number of atypical sites: the same location shift parameter value case (*N* = 48). The planned number of patients at the final CSM analysis of 48 is used. The values of location shift parameters are shown on the top of the panel and The ID's of atypical sites are shown on the left side of the panel. The lines in each graph show the cumulative detection probability of atypical sites by CSM analysis and type I error averaged over the normal sites. The upper graphs show the case where only one atypical site exists. The middle graphs show the case in which two atypical sites exist and the lower graphs show that three atypical sites exist.

**Fig. 4:**
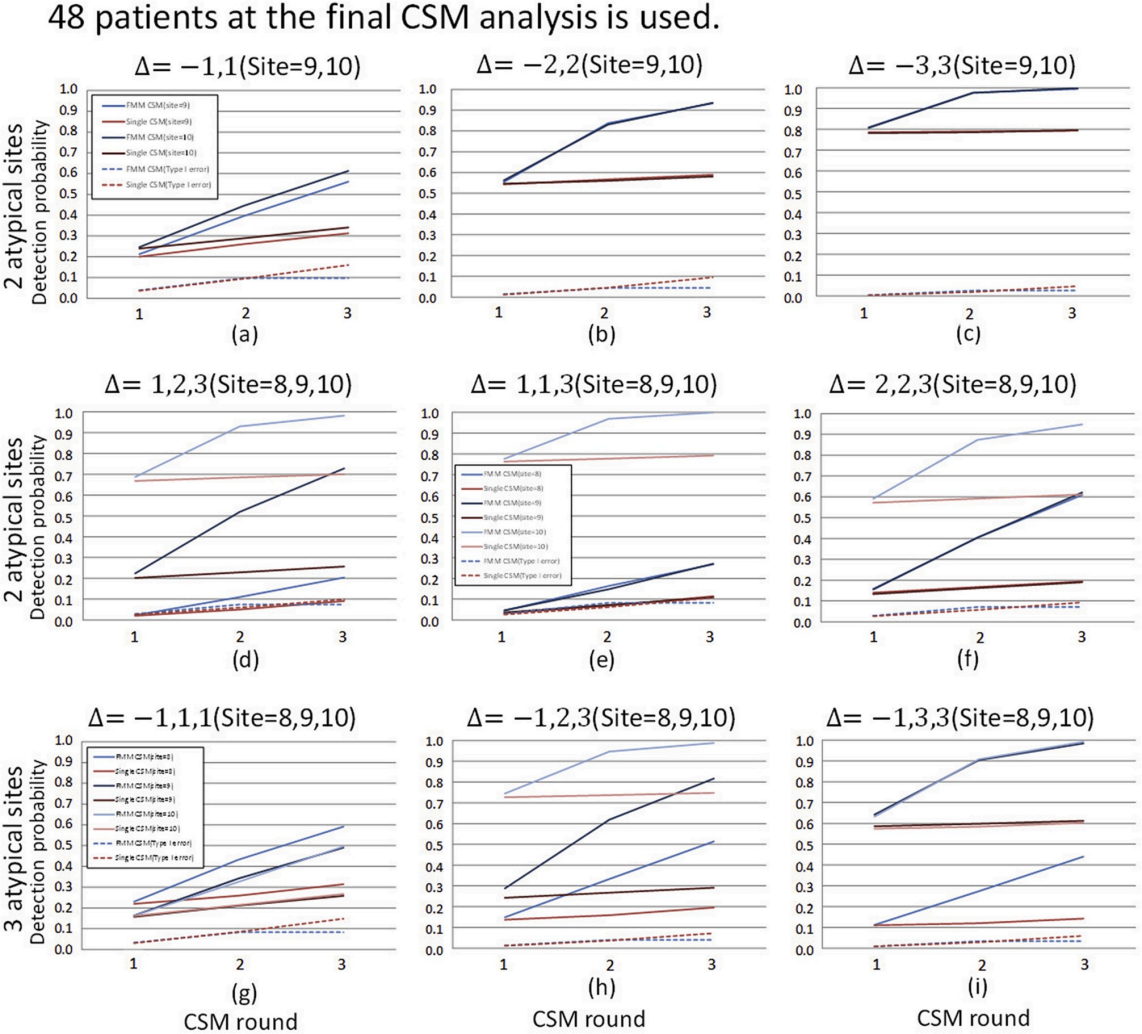
Cumulative detection probability stratified by location shifts and the number of atypical sites: different location shift parameter value case (*N* = 48). The planned number of patients at the final CSM analysis of 48 is used. The values of location shift parameters and the site ID's of atypical sites are shown on the top of each graph. The other display formats are the same as those in Fig. 3.

**Fig. 5:**
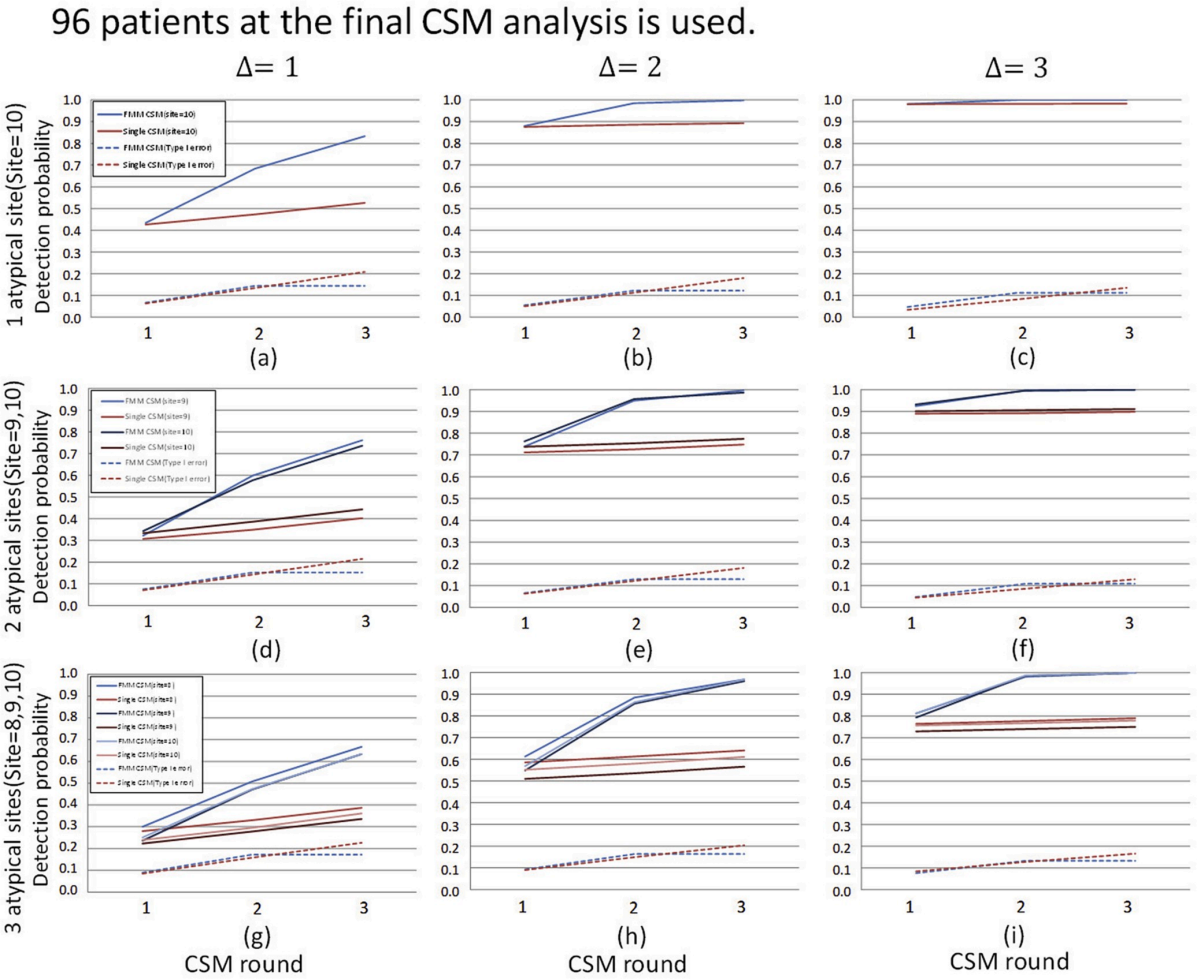
Detection probability stratified by location shifts and the number of atypical sites: the same location shift parameter value case (*N* = 96). The planned number of patients at the final CSM analysis of 96 is used. The other display formats are the same as those in Fig. 3.

**Fig. 6:**
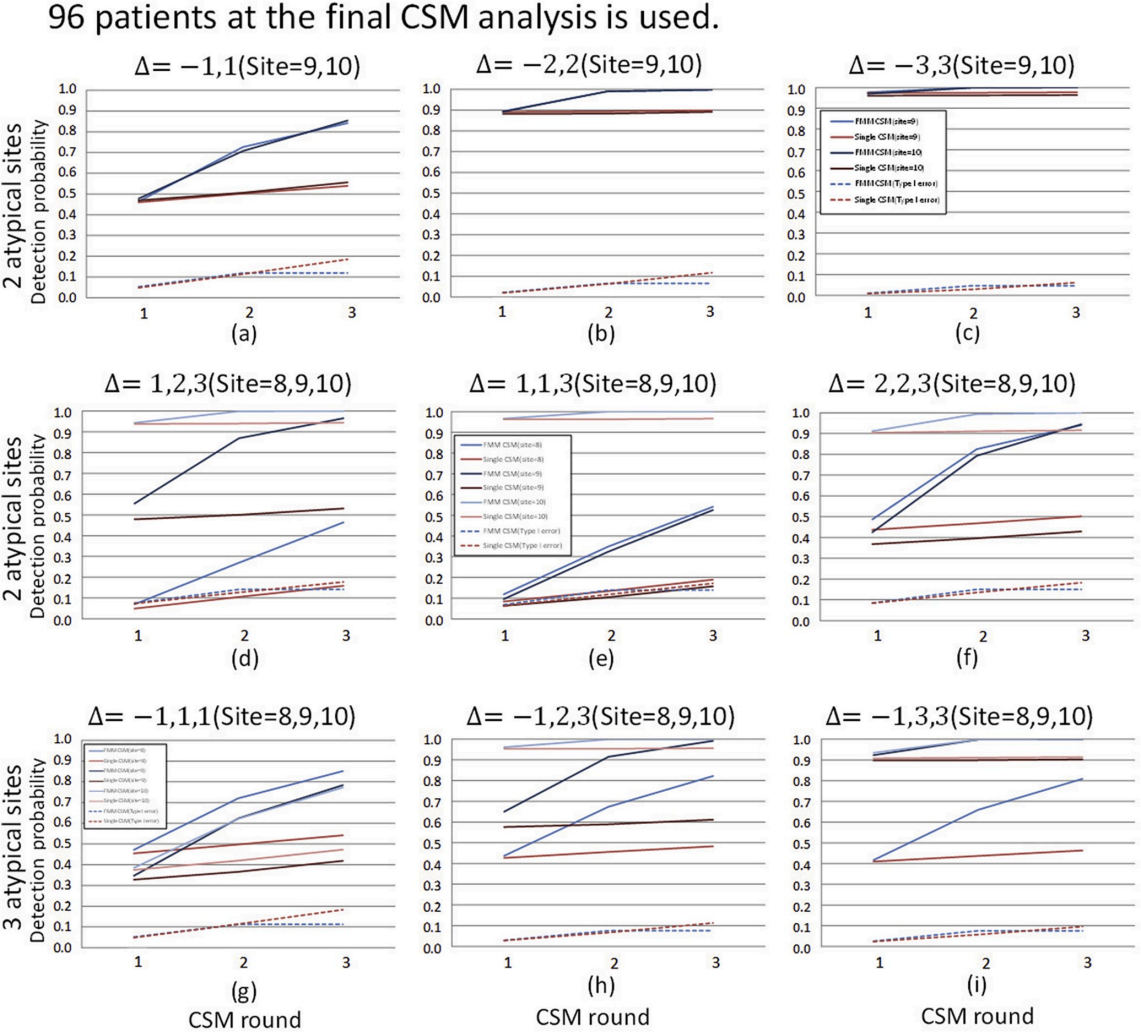
Detection probability stratified by location shifts and the number of atypical sites: different location shift parameter value case (*N* = 96). The planned number of patients at the final CSM analysis of 96 is used. The other display formats are the same as those in Fig. 3.

As described in Section 2.1, in the t-th analysis of CSM, the statistical model is estimated based on the outcome Y(t) which is observed by the t-th analysis of CSM, and the posterior predictive distribution is updated whereas ${\bar{y}}_{i}\left(t\right)$ is calculated using the outcomes $y_{i}\left(t\right)$ which is newly observed after the (t−1)-th analysis $\left(y_{i}\left(t\right)=Y_{i}\left(t\right)＼Y_{i}\left(t-1\right)\;\;t>1,\;\;\;y_{i}\left(1\right)=Y_{i}\left(1\right)\;\;\;t=1\right)$. In addition, the sites detected as atypical are investigated by on-site monitoring and it is assumed that the operational processes of the sites are changed to the state of normal by corrective action. Therefore, even in the sites detected as atypical, data after detection follows the same distribution as in the normal sites. Without loss of generality, we assume that patient outcomes in normal or atypical sites independently follow the identical $N\left(0,1^{2}\right)$ or $N\left(\text{Δ},1^{2}\right)$, respectively. We evaluated detection performance in the various combinations of location shift parameter Δ and the number of the atypical sites. The results are shown in Fig. 3, Fig. 4, Fig. 5, Fig. 6.

In both single CSM and FMM-CSM, we assume non-informative prior distributions N(0,1000) and InvGamma(0.1,0.1) for mean and variance, respectively. In FMM-CSM, taking into account the possibility that location shift of data from atypical sites are occurred in both lower and upper sides, we set the number of components of the FMM model to K=3. For a prior distribution of mixture parameters, which is informative, we assume $\pi_{k}\sim Dirichlet\left(1,8,1\right)$ based on the assumption that normal sites are a majority. As the threshold for atypical sites detection, α=0.05 is used for both Eqs. (5) and (9).

In all scenarios, the simulation is repeated 1000 times by SAS for Windows release 9.4 (SAS Institute Inc., Cary, NC, USA). The posterior distribution is calculated by Markov Chain Monte Carlo method of SAS FMM procedure. The main analysis code can be found in Appendix A. The simulation run time for 1000 repetitions was about 18 h in our computational environment (OS: Windows 10 64bit, CPU: 1.7 GHz, RAM: 8.0 GB).

### 4.2 Results

FMM-CSM method is evaluated by the measures of the cumulative detection probability of atypical sites and the cumulative type I error averaged over normal sites for some scenarios varying the number of abnormal sites and the values of location shift parameter Δ. It is defined that the cumulative detection probability is the ratio that true atypical site is detected as atypical until the *t*-th round of CSM. It is defined that the cumulative type I error averaged over normal sites is the average of cumulative type I error ratios which are calculated by truly normal sites, where the cumulative type I error is the occurrence of type I error until *t*-th round of CSM. Since both evaluation measures are the estimated probabilities, the results from Monte Carlo simulation follows the binominal distribution with the number of simulation repetitions and either the cumulative detection probability or the cumulative type I error. Let $N_{sim}$, $\hat{\pi }$ be simulation repetitions and either the calculated cumulative detection probability or the calculated cumulative type I error, respectively. Hence, a simulation error for the cumulative detection probability of atypical sites is approximately $\sqrt{\hat{\pi }\left(1-\hat{\pi }\right)/N_{sim}}$ as the standard deviation. For the cumulative type I error averaged over normal sites, a simulation error is $\sqrt{\hat{\pi }\left(1-\hat{\pi }\right)/N_{sim}N_{normal}}$ where $N_{normal}$ is the number of normal sites.

Fig. 3 shows the result of N=48 case. In these scenarios, the values of location shift parameter Δ among the atypical sites are the same values. The results show that the detection probabilities of single CSM and FMM-CSM are almost the same at the first analysis of CSM, however, the difference in detection probabilities between the methods becomes larger as CSM is sequentially performed. The results confirm the fact explained in Section 2.2 that single CSM is considerably affected by data from atypical sites, which considerably decreases its detection performance, in particular, in the case where the proportion of atypical sites is large. On the other hand, our method is able to detect atypical sites regardless of the proportion of atypical sites. In addition, the type I error averaged over the normal sites does not show a considerable difference between the methods. Moreover, FMM-CSM outperforms single CSM substantially in detection probability as the proportion of atypical sites increases. In practical clinical trials, the distributions of outcomes are not necessarily the same among the atypical sites. The location shift in the distribution of outcomes may appear on both upper and lower sides in some cases, or it may appear on the same side in other cases, and moreover, it may occur with different shift sizes. In the cases where the sizes of location shifts are different among atypical sites, it is of concern that the detection performance for the atypical sites in which the size of location shifts in the outcome distribution is moderate are considerably decreased for a single distribution-based method. The statistical models used in the single distribution-based methods are affected considerably by the data from the atypical sites in which the differentiation of location shift sizes is large among sites, and it leads to overlooking smaller location shifts. However, the severity of the process abnormality may not be directly reflected in the size of the location shift, and the decline in detection performance for the atypical sites with moderate location shifts should not be neglected.

To evaluate detection performance in the above cases, we conduct simulations in case where the values of location shift parameter Δ are heterogeneous. The results are shown in Fig. 4. Fig. 4(a)–(c) show the results of the case where the location shifts in atypical sites occur on both sides. The results show that FMM-CSM is able to detect atypical sites with higher probabilities in any cases with keeping at the same level as type I error as of single CSM. Fig. 4(d)–(f) show the results of the cases where the location shifts occur in the same direction. It is shown that the detection probabilities for the atypical sites with moderate location shift are lowered especially in single CSM. On the other hand, it is shown that proposed FMM-CSM is able to detect not only the atypical sites with large location shifts but also the atypical sites with moderate location shifts with higher probabilities than those of single CSM. Fig. 4(g)–(i) show the results of the cases that location shifts with various sizes appear in both sides and the number of atypical sites is not balanced on each side. In those cases, it is shown that FMM-CSM detects atypical sites with higher probabilities than those of single CSM with keeping at the same level as type I error as of single CSM.

To evaluate how much the number of patients affects the detection performance in CSM methods, the simulation results of the cases where N=96 are shown in Fig. 5, Fig. 6. Comparing the detection probabilities between the results of N=48 and N=96 within the same scenario, it is shown that the magnitude of improvement of detecting performance by FMM-CSM is relatively larger in the results of N=48 (the small sized trial cases) than in the results of N=96. For instance, Fig. 4, Fig. 6 are the results of N=48 and N=96 in the same scenario, respectively. In Fig. 4(d), the cumulative detection probabilities of site 9 at the final analysis are 0.257 in single CSM and 0.728 in FMM-CSM, and the ratio is 2.83. The results show that FMM-CSM is able to detect atypical sites with higher probabilities in any cases with keeping at the same level as type I error as of single CSM as with N=48 cases.

Even though the number of CSM analyses may be determined by taking account of operational feasibility, it is important to know how much frequency of the CSM analysis affects the performance of CSM to choose the number of CSM analyses. Therefore, we examined the detection performance of CSM in the situations where the CSM analysis was conducted one or two times. The simulation results are shown in Appendix B of Figs. S1–S8. As expected it is confirmed that the detection probability by the final analysis becomes higher as the number of CSM analyses increase. In practical CSM, if an abnormality is detected at a certain time, site inspection including on-site monitoring is performed and the operational process is corrected. After that, normal outcomes are observed from the relevant sites. Therefore, if an abnormality is detected at an early stage of a clinical trial, then the normal data which can be used for model estimation increases in the subsequent CSM. Accordingly, the detection probabilities by the final analysis become high as the number of CSM analyses increase.

In this article, we assume that the normal sites are majority. However, it is important to examine the performance of proposed method in the extreme cases of which the sites to be detected are the same proportion as the normal sites, and the case of which only normal sites exist to evaluate the robustness of the method against extreme situations. We conduct simulation studies of both cases. The results are shown in Appendix B of Figs. S9 and S10. These results show that FMM-CSM is able to detect the sites to be detected with slightly higher probability than those of single CSM while maintaining Type I error as much as singe CSM even in the cases of the proportions of the normal sites and the sites to be detected is the same. In addition, it is shown that the type I error averaged over the normal sites of the FMM-CSM is as much as those of single in the cases of all sites are normal.

In this study, we use Dirichlet(1,8,1) as a prior of mixture parameters. In addition, to examine the effect of a prior distribution of mixture parameters, we conduct sensitivity analysis via simulation in cases whereDirichlet(0.5,9,0.5) is used. The simulation results are shown in Appendix B of Figs. S11–S14. There is less difference between the results of Dirichlet(0.5,9,0.5) prior and Dirichlet(1,8,1) prior cases, and FMM-CSM is robust against the selection of a prior distribution as long as using the prior distribution based on the assumption that the normal sites are a majority. Moreover, we conducted simulation in the case of Dirichlet(1,1,1) used for a sensitivity analysis (results not shown), but as expected, the FMM-CSM does not work effectively. However, the application of such a prior distribution cannot be considered in real CM, because CSM is premised on being implemented in well-processed controlled clinical trials. It should be assumed the sites to be detected are a minority. If it is considered that such a prior distribution should be used, the process control of the clinical trial itself should be reviewed.

## 5 Example of the realistic CSM

We illustrate the artificial central statistical monitoring using a Bayesian FMM-CSM. Five sites participate in a multicenter clinical trial and the analysis of CSM performs twice. The 38 patients in total are enrolled and outcomes are continuous variables which independently follow identical normal distribution. There is an atypical site (site 5) at the first round of the CSM, and remaining sites (sites 1–4) are typical. We assume that all the sites have been already opened and the timing of patient enrollments is random. Thus, the number of patients for each site is unbalanced over sites.

Table 1 shows all the outcomes from this clinical trial. In the first round of CSM, we use data as follows:

$Y_{1}\left(1\right)=y_{1}\left(1\right)=\left(5.2,4.8\right)$, $Y_{2}\left(1\right)=y_{2}\left(1\right)=\left(5.5,5.2,5.0\right)$
$Y_{3}\left(1\right)=y_{3}\left(1\right)=\left(4.7,5.5,5.5,5.0,5.8\right)$, $Y_{4}\left(1\right)=y_{4}\left(1\right)=\left(5.1,5.0,6.0,4.8,5.6\right)$
$Y_{5}\left(1\right)=y_{5}\left(1\right)=\left(6.3,5.3,6.5,6.2\right)$.

**Table 1:** Artificial outcomes, site averages, and critical limits in the first round of CSM.

The first round																			
Site	1	1	2	2	2	3	3	3	3	3	4	4	4	4	4	5	5	5	5
outcomes	5.2	4.8	5.5	5.2	5.0	4.7	5.5	5.5	5.0	5.8	5.1	5.0	6.0	4.8	5.6	6.3	5.3	6.5	6.2
Average	5.0		5.2			5.3					5.3					6.1			
critical limits	[4.7, 6.1]		[4.9, 6.0]			[5.0, 5.8]					[5.0, 5.8]					[4.9, 5.9]			

The body distribution is estimated for the above data through Bayesian FMM with the same settings of prior distributions as section 4.2. As a result, the component with the largest expectation in the posterior distributions of the mixture parameters $\left(\pi_{1},\pi_{2},\pi_{3}\right)$ is the second component, i.e., $E\left[\left(\pi_{1},\pi_{2},\pi_{3}\right)\right]=\left(\;0.036,\;0.926,\;0.037\right)$. Then, from the posterior predictive distribution for each site average, critical limits, $\gamma_{i}^{0.05}\left(1\right),\;\gamma_{i}^{0.95}\left(1\right)$ for each site can be calculated and they are shown in the bottom line in Table 1. On the other hand, actual site average for each site is shown in the second line from the bottom in Table 1. By comparing actual site average with critical limits, we can predict that the site 5 is atypical because of out of critical limits. We assume that the site 5 turns into being normal by succeeding to corrective actions for root causes generating atypical operation processes in the site 5.

Table 2 shows all the outcomes at the 2nd round of CSM. In the second round of CSM, data for specifying a body distribution are as follows:

$$Y_{1}\left(2\right)=\left(y_{1}\left(1\right),y_{1}\left(2\right)\right)=\left(5.2,\;4.8,\;5.1,\;5.0,\;5.3,\;5.3,\;5.1,\;4.6\right)$$

$$Y_{2}\left(2\right)=\left(y_{2}\left(1\right),y_{2}\left(2\right)\right)=\left(5.5,\;5.2,\;5.0,\;5.4,\;5.4,\;4.9,\;5.5\right)$$

$$Y_{3}\left(2\right)=\left(y_{3}\left(1\right),y_{3}\left(2\right)\right)=\left(4.7,\;5.5,\;5.5,\;5.0,\;5.8,\;5.4,\;5.3,\;5.5\right)$$

$$Y_{4}\left(2\right)=\left(y_{4}\left(1\right),y_{4}\left(2\right)\right)=\left(5.1,\;5.0,\;6.0,\;4.8,\;5.6,\;6.0,\;5.8\right)$$

$$Y_{5}\left(2\right)=\left(y_{5}\left(1\right),y_{5}\left(2\right)\right)=\left(6.3,5.3,6.5,6.2,5.3,5.4,5.5,5.6\right)$$

**Table 2:** Artificial outcomes, site averages, and critical limits in the second round of CSM.

The 2nd round																			
site	1	1	1	1	1	1	2	2	2	2	3	3	3	4	4	5	5	5	5
outcomes	5.1	5.0	5.3	5.3	5.1	4.6	5.4	5.4	4.9	5.5	5.4	5.3	5.5	6.0	5.8	5.3	5.4	5.5	5.6
average	5.1						5.3				5.4			5.9		5.5			
critical limits	[5.1, 5.7]						[5.0, 5.8]				[4.9, 5.8]			[4.8, 5.9]		[5.0, 5.7]			

The body component is specified as the second one, i.e., $E\left[\left(\pi_{1},\pi_{2},\pi_{3}\right)\right]=\left(\;0.024,\;0.951,\;0.025\right)$. As the same manner as the first round of CSM, critical limits, $\gamma_{i}^{0.05}\left(2\right),\;\gamma_{i}^{0.95}\left(2\right)$ for each site and site averages are shown at the first and the second lines of Table 2, respectively. Note that the site average of the i-th site is calculated from only data $y_{i}\left(2\right)$. We can recognize that none of site is atypical.

## 6 Discussion

In this study, we propose the FMM-CSM method using Bayesian Finite Mixture Models as the method which detects the atypical site with high probability regardless of the proportion of the atypical sites intended to use in small clinical trials and the beginning of the trials. The existing CSM methods are based on the assumption that the number of clinical sites is not small and the proportion of atypical sites is small.

In these existing methods, it is justified to use a statistical model based on single distribution without accounting for contamination of the data from atypical sites based on the assumption. However, as our targeted situations, the implementation of CSM is necessary in small sized trials and the beginning of the trials with no large number of clinical sites. Moreover, the abnormalities in operational processes in the sites occur for a wide variety of cases, such as a misunderstanding of study protocols and a mistuning of measurement equipment, and it is not always reasonable to assume that the proportion of sites to be detected is small. Thus, if the single distribution-based method is used in practical clinical trials, it may lead to a decline of detection performance due to contamination of the data from atypical sites. As a result, overlooking of serious process abnormalities increases. Actually, in our simulation study, it is shown that the detection performance considerably decreases as the proportion of atypical sites increases if single distribution-based methods are used. However, it is shown that our method detects atypical sites with high probability regardless of the proportion of the atypical sites under the small clinical trial settings which is the target of our proposed method.

In actual clinical trials, CSM is performed sequentially and inspection through use of on-site monitoring is conducted if the signals of abnormalities are detected, and operational processes of the relevant sites are corrected. Therefore, the number of atypical sites and the proportion of the data from atypical sites are expected to decrease. The detection performance of CSM methods depend not only on the amount of data but also on the frequency of analysis. However, the performance of CSM with multiple analyses have not been investigated sufficiently so far. In this study, we evaluate the performance of our method when the analysis is multiply conducted with the progress of the trials.

Failure to detect the signals of process abnormalities by CSM is serious problem related to data quality. Especially in small clinical trials, the data from one abnormal site will affect analysis. In this study, the statistical multiplicity of comparisons was not considered because authors think that it is feasible to take priority to detect process abnormalities over maintaining false positive rate in the CSM of such small clinical trials. However, even in the small clinical trials, the overly large false positive rate invoked the ineffective inspection by on-site monitoring should be avoided. It is possible to control familywise type I error rate by adjusting the threshold value of detection, αthrough simulation studies. It is also possible to control familywise type I error rate formally by using the multiple comparison procedure such as closed procedure for comparison of multiple sites, and control the familywise type I error rate attributed to CSM's multiple round by using group sequential methods such as alpha spending functions [26]. However, we should consider carefully doing that because it may lead to a decline of the detection performance of an individual atypical site.

In this study, we consider the case where the number of sites is not so large, and we adapted not complicated model whose component number is three. The component number is minimum number to take account the location shift in outcome distributions may occurs in both lower and upper sides. When the number of sites is large, it is also useful to select the optimal number of components K automatically using information criteria such as AIC and BIC [22,23], or the methods to model the number of components is available [[22], [23], [24],^27^]. Moreover, model averaging technique [28] may be useful for improving fitness of the model to the data. However, improving the fitness of the model to the data may not necessarily lead to a better detection performance. In practice, we recommend setting up a model used in analysis via simulation studies that takes into account of what outcomes are analyzed, how much sites are, how frequently abnormalities occur, or how different of the outcomes from atypical sites are, etc.

In this study, we assume that outcomes are normally distributed continuous value such as HbA1c value and blood pressure, and consider the detection of the sites whose outcome distributions have location shifts for simple discussion. In practical clinical trials, the scale parameter of the outcome distributions in atypical sites may have a different tendency from other sites. In addition, not only continuous values but also discrete values including adverse events counts and time to event including time to withdrawal may be analyzed in actual CSM. For analyzing these various types of data, the statistical model of the proposed method is needed to be changed to according to the data type such Poisson regression model for count data, Weibull distribution model for time to event data and beta-binomial model for proportion which used in Desmet et al. [16]. To adopt these models for our proposed method based on FMM, it is also necessary to use MCMC for estimation. The expansion of our method for different type of variable is our future work.

We proposed a tractable single variable based method assuming normal distribution within each compartment because our focus point is the proposal for the CSM method which is the robust against the existence of atypical sites in small clinical trials or the beginning of clinical trials. However, in large clinical trials, it might be more useful to use multiple variables simultaneously for detecting atypical sites. Currently, the Hotelling's *T*^2^ statistic (the Mahalanobis distance) is used to detect multivariate outliers in CSM [9]. Desmet et al. [15] discussed the detection procedure with combining the *p*-values obtained for many variables to detect atypical sites (ensemble learning). However, it is difficult to investigate the causes that the site is out of normal. Mason et al. [17] and Zink et al. [18] tackle the problem of investigating the contribution of the individual variables to each multivariate outlier after detection of abnormalities. Zink et al. [18] illustrated the application of the contribution plot [19] to RBM for real multicenter clinical trials. Mason et al. [17] proposed to calculate the magnitude of the contribution using decomposition of Hotelling's *T*^2^ statistic into one dimensional variate. Though the decomposition or the contribution plots help us recognizing which variables shifted, it does not directly tell us what the root causes are because variables monitored are outcomes. In general, variables have correlations each other due to causality. Hence, multivariate monitoring is more complicated than univariate monitoring. In actual, in order to efficiently success finding and eliminate root causes, we would need to identify the cause and effect relationship beforehand or each round of CSM. In addition, the analysis methods would require the countermeasure of resistance against outliers to estimate an adequate variance-covariance matrix, which is to apply robust estimators for statistical process control based on minimum volume ellipsoid, minimum covariate determinant [29]. However, using complicated methods might be computationally intractable and might need more powerful computational environment such as cloud computational service. Thus, in practice, atypical site detection by multivariate monitoring would be controversial in CSM.

To use our method in those various cases, it may be necessary to adopt a model to analyze outcomes appropriately. Though further research is required to construct the optimal method for each type of outcome, it is the only a problem of model selection, and proposed methods can be extended to these various situations of clinical trials. In this study, we proposed FMM-CSM method as a Bayesian method which allows adaptation of informative prior distribution. By using Bayesian approach, the possibility of process abnormality is displayed as probabilities of the sites are different from normal site, it is intuitively understandable and easy to explain to non-statistician such as clinical research associates and investigators. In the simulation of this study, we used informative prior for mixture parameter based on the assumption of normal sites are majority. Non-informative priors are used for location and scale parameters, however it is possible to use informative priors from the historical data of the studies in the same therapeutic areas. If appropriate informative prior is used, the method may be more effective especially in the small clinical trials and the beginning of the trials, which are targets.

In this study, it is assumed that the detected atypical sites turn into normal sites immediately. This assumption may be not truly strictly realistic. However, this assumption is the ideal situation that we have to achieve in RBM. From the viewpoint of process control, the principal concept of central statistical monitoring is the attempt that atypical sites are surely corrected by focusing on only a few sites detected and intensively assigning resource instead of monitoring all the sites equally at once. The performance of our method for the case of violating this assumption will evaluated through extensive simulation in the future work.

## Funding

This research did not receive any specific grant from funding agencies in the public, commercial, or not-for-profit sectors.

## Declaration of competing interest

The Authors declare that there is no conflict of interest.

## Appendix Supplementary data

Appendix A Appendix A (SAS code)
