# Supplementary material for: Bayesian central statistical monitoring using finite mixture models in multicenter clinical trials
Source: Contemp Clin Trials Commun. 2020 Apr 9;19:100566. doi: 10.1016/j.conctc.2020.100566 (PMC7358264; doi:10.1016/j.conctc.2020.100566)
Supplement: Appendix A [file mmc1.pdf]

```
/*analysis using FMM  MCMC sampling from posterior dist.*/
proc fmm data=dat; /* data set input */
    model val= / dist=normal k=3;
     bayes mupriorparms=(0,1000) phipriorparms=(0.1, 0.1) MIXPRIORPARMS(1,8,1)  nbi=50000 initial=random
     nmc=400000 thin=50 outpost=out ;
run;

/* ----choose the component whose posterior mean of mixture parameter is the largest as body distribution*/
data pout;
    set out;
    p1=Parm_7;
    p2=Parm_8;
    p3=1-p1-p2;
    keep p1 p2 p3;
run;
proc means data=pout;
    var p1;
    output out=poutm1 mean=mean;
run;
proc means data=pout;
    var p2;
    output out=poutm2 mean=mean;
run;
proc means data=pout;
    var p3;
    output out=poutm3 mean=mean;
run;
data _null_;
    set poutm1;
    call symput("p1", put(mean, best.-l));
run;
data _null_;
    set poutm2;
    call symput("p2", put(mean, best.-l));
run;
data _null_;
```

```
    set poutm3;
    call symput("p3", put(mean, best.-l));
run;
data model;
    do repm=1 to 3;
          if repm=1 then p=&p1.;
          if repm=2 then p=&p2.;
          if repm=3 then p=&p3.;
          rep=1;
          output;
    end;
run;
proc sort data=model;
    by descending p;
run;
data model;
    set model;
    by rep;
    if first.rep;
run;
data _null_;
    set model;
       /* macro parameter "model" includes the component number of body distribution*/
    call symput("model", put(repm,best.-l));
run;
/*------------------------------------------------------------*/

data out;
    set out;
    if &model.=1 then mu=Parm_1;
    if &model.=2 then mu=Parm_2;
    if &model.=3 then mu=Parm_3;
    if &model.=1 then V=Parm_4;
    if &model.=2 then V=Parm_5;
    if &model.=3 then V=Parm_6;
    SD=V**0.5;
```

```
run;
/*count site number*/
proc sort data=dat out=num nodupkey;
    by site;
run;
data _null_;
    set num nobs=n;
    call symput("site_no", put(n, best.-l));
run;
%macro loop();
%do k=1 %to &site_no.;/*set the number of sites*/
    data _null_;
          set dats_m;
          if site=&k. then call symput("repn_&k.", put(n, best.-l) );
    run;
    /*----posterior predictive destribution of site mean-----*/
    data pred_&k.;
          set out(keep=mu sd);
           do j=1 to &&repn_&k.;
               Y_pred=rand("normal", mu, sd);
               rand=rand("uniform");
               output;
         end;
          run;
          proc sort data=pred_&k.;
              by rand;
          run;
          data pred_&k.;
              set pred_&k.;
              rep+1;
              if rep>&&repn_&k. then rep=1;
          run;
          data pred_&k.;
              set pred_&k.;
              if rep=1 then repn+1;
          run;
```

```
          proc means data=pred_&k. noprint;
              by repn;
              var y_pred;
              output out=predM&k. mean=Y_predM;
          run;
          /*---------------------------------------------------*/
          data predM&k.;
              set predM&k.;
              site=&k.;
          run;
%end;
%mend loop;
%loop;

data predM;
    set predM1-predM&site_no.;
run;
/*prediction interval of the site means*/
proc means data=predM;
    by site;
    var Y_predM;
    output out=pred_th p5=p5_predth p95=p95_predth;
run;
/*--------calculate site mean-------------------------------------*/
proc sort data=dat;
    by site;
run;
proc means data=dat;
    by site;
    var val;
    output out=dats_m n=n mean=mean;
run;
/*-----------------------------------------------------------------*/

/*Detection of atypical sites*/
data dats_d;
```

```
    merge dats_m pred_th;
    by site;
    if .z< mean < p5_predth  then flg=1;  /*flagging the atypical sites*/
    if p95_predth<mean       then flg=1;
run;
```
