# Supplementary material for: Bayesian central statistical monitoring using finite mixture models in multicenter clinical trials
Source: Contemp Clin Trials Commun. 2020 Apr 9;19:100566. doi: 10.1016/j.conctc.2020.100566 (PMC7358264; doi:10.1016/j.conctc.2020.100566)
Supplement: Appendix [file mmc2.docx]

Figure S1. The result of the case that CSM is conducted only one time. Detection probability stratified by location shifts and the number of atypical sites: the same location shift parameter value case (N=48). The values of location shift parameters are shown on the top of the panel and The ID’s of atypical sites are shown on the left side of the panel. The bars in each graph show the cumulative detection probability of atypical sites by CSM analysis and type I error averaged over the normal sites. The upper graphs show the case where only one atypical site exists. The middle graphs show the case in which two atypical sites exist and the lower graphs show that three atypical sites exist.

Figure S2. The result of the case that CSM is conducted only one time. Detection probability stratified by location shifts and the number of atypical sites: deferent location shift parameter value case (N=48). The values of location shift parameters and the site ID’s of atypical sites are shown on the top of each graph. The other display formats are the same as Figure S1.

Figure S3. The result of the case that CSM is conducted two times. Detection probability stratified by location shifts and the number of atypical sites: the same location shift parameter value case (N=48). The values of location shift parameters are shown on the top of the panel and The ID’s of atypical sites are shown on the left side of the panel. The lines in each graph show the cumulative detection probability of atypical sites by CSM analysis and type I error averaged over the normal sites. The upper graphs show the case where only one atypical site exists. The middle graphs show the case in which two atypical sites exist and the lower graphs show that three atypical sites exist.

Figure S4. The result of the case that CSM is conducted two times. Detection probability stratified by location shifts and the number of atypical sites: deferent location shift parameter value case (N=48). The values of location shift parameters and the site ID’s of atypical sites are shown on the top of each graph. The other display formats are the same as Figure S3.

Figure S5. The result of the case that CSM is conducted only one time. Detection probability stratified by location shifts and the number of atypical sites: the same location shift parameter value case (N=96). The values of location shift parameters and the site ID’s of atypical sites are shown on the top of each graph. The other display formats are the same as Figure S1.

Figure S6. The result of the case that CSM is conducted only one time. Detection probability stratified by location shifts and the number of atypical sites: deferent location shift parameter value case (N=96). The values of location shift parameters and the site ID’s of atypical sites are shown on the top of each graph. The other display formats are the same as Figure S1.

Figure S7. The result of the case that CSM is conducted two times. Detection probability stratified by location shifts and the number of atypical sites: the same location shift parameter value case (N=96). The values of location shift parameters and the site ID’s of atypical sites are shown on the top of each graph. The other display formats are the same as Figure S3.

Figure S8. The result of the case that CSM is conducted two times. Detection probability stratified by location shifts and the number of atypical sites: deferent location shift parameter value case (N=96). The values of location shift parameters and the site ID’s of atypical sites are shown on the top of each graph. The other display formats are the same as Figure S3.

Figure S9. The result of the case that CSM is conducted three times. Detection probability stratified by location shifts and the number of atypical sites: the same location shift parameter value case (N=48). The values of location shift parameters are shown on the top of the panel and the numbers of atypical sites are shown on the left side of the panel. The lines in each graph show the cumulative detection probability averaged over the normal sites by CSM analysis and type I error averaged over the normal sites. The upper graphs show the case where five atypical sites exists. The lower graphs show the case in which no atypical sites exist.

Figure S10. The result of the case that CSM is conducted three times. Detection probability stratified by location shifts and the number of atypical sites: the same location shift parameter value case (N=96). The values of location shift parameters are shown on the top of the panel and the numbers of atypical sites are shown on the left side of the panel. The lines in each graph show the cumulative detection probability averaged over the normal sites by CSM analysis and type I error averaged over the normal sites. The upper graphs show the case where five atypical sites exists. The lower graphs show the case in which no atypical sites exist.

Figure S11. The result of the sensitivity analysis that $Dirichlet\left( 0.5,9,0.5 \right)$ is used as the prior distribution of mixture parameter of FMM. Detection probability stratified by location shifts and the number of atypical sites: the same location shift parameter value case (N=48). The values of location shift parameters are shown on the top of the panel and The ID’s of atypical sites are shown on the left side of the panel. The lines in each graph show the cumulative detection probability of atypical sites by CSM analysis and type I error averaged over the normal sites. The upper graphs show the case where only one atypical site exists. The middle graphs show the case in which two atypical sites exist and the lower graphs show that three atypical sites exist.

Figure S12. The result of the sensitivity analysis that $Dirichlet\left( 0.5,9,0.5 \right)$ is used as the prior distribution of mixture parameter of FMM. Detection probability stratified by location shifts and the number of atypical sites: deferent location shift parameter value case (N=48). The values of location shift parameters and the site ID’s of atypical sites are shown on the top of each graph. The other display formats are the same as Figure S11.

Figure S13. The result of the sensitivity analysis that $Dirichlet\left( 0.5,9,0.5 \right)$ is used as the prior distribution of mixture parameter of FMM. Detection probability stratified by location shifts and the number of atypical sites: the same location shift parameter value case (N=96). The values of location shift parameters and the site ID’s of atypical sites are shown on the top of each graph. The other display formats are the same as Figure S11.

Figure S14. The result of the sensitivity analysis that $Dirichlet\left( 0.5,9,0.5 \right)$ is used as the prior distribution of mixture parameter of FMM. Detection probability stratified by location shifts and the number of atypical sites: deferent location shift parameter value case (N=96). The values of location shift parameters and the site ID’s of atypical sites are shown on the top of each graph. The other display formats are the same as Figure S11.
